# Acute Acalculous Cholecystitis Due to a Primary Epstein Barr Virus Infection in a Pediatric Patient

**DOI:** 10.3390/pediatric13010011

**Published:** 2021-02-06

**Authors:** Carlos Leganés Villanueva, Ilaria Goruppi, Nuria Brun Lozano, Federica Bianchi, María Quinteiro González, Susana Boronat Guerrero

**Affiliations:** 1 Pediatric Surgery Unit, Department of Pediatrics, Hospital de la Santa Creu i Sant Pau, Universitat Autònoma de Barcelona, 08041 Barcelona, Spain; igoruppi@santpau.cat (I.G.); nbrun@santpau.cat (N.B.L.); fbianchix@santpau.cat (F.B.); 2 Pediatric Unit, Department of Pediatrics, Hospital de la Santa Creu i Sant Pau, Universitat Autònoma de Barcelona, 08041 Barcelona, Spain; mquinteiro@santpau.cat (M.Q.G.); sboronat@santpau.cat (S.B.G.)

**Keywords:** acute acalculous cholecystitis, Epstein Barr virus, conservative management, infectious mononucleosis, pediatrics

## Abstract

Epstein–Barr virus (EBV) is estimated to infect more than 98% of adults worldwide and is one of the most common human viruses. Acute acalculous cholecystitis (AAC) of the gallbladder is an atypical complication of infectious mononucleosis caused by EBV. Conservative management has been described in the context of AAC caused by EBV. A surgical approach must be considered in the case of acute complications such as perforation or gallbladder gangrene. We present the case of a 10-year-old female patient with AAC due to infectious mononucleosis syndrome caused by primary EBV infection.

## 1. Introduction

Acute acalculous cholecystitis (AAC) is a gallbladder infection not related to gallstones, leading to serious consequences. AAC is caused by gallbladder stasis from hypomotility that leads to increased intraluminal pressures in the gallbladder wall, resulting in ischemia, inflammation, and potential necrosis [1]. Only 5–10% of acute cholecystitis cases are AAC in adults, and they have higher morbidity and mortality than calculous cholecystitis. Acute inflammatory gallbladder diseases including ACC are relatively uncommon in pediatric patients [2].

Acute infection with Epstein–Barr virus (EBV) is a common disease mainly affecting adolescents and young adults and it is usually asymptomatic; it often presents as typical infectious mononucleosis symptoms such as fever, sore throat, cervical lymphadenopathy, and hepatosplenomegaly. Hepatic involvement is very common and AAC may occur during acute primary EBV infection, which is rarely encountered in the pediatric population [3].

To date, only 24 cases of AAC caused by EBV infection have been reported in pediatrics [4]. In this report, we present a case of primary EBV infection complicated by AAC in a 10-year-old patient.

## 2. Case Report

A 10-year-old female was brought to the emergency department complaining of a 7-day history of low-grade fever up to 38.5 °C, sore throat, mild cough, sharp abdominal pain, mainly in the right abdominal area, and nausea. Physical examination revealed sensitivity to palpation in the right hypochondriac and lumbar regions and epigastrium, normal bowel sounds, and no abdominal distention.

Additionally, bilateral cervical and axillary lymphadenopathy, tonsillar hypertrophy without exudates, and a palpable hepatosplenomegaly were noticed, and there was no conjunctival icterus.

Laboratory studies showed a white blood cell count of 7200 × 10^3^ per microliter (μL) (4.5–14.5 × 10^3^/μL), a C-reactive protein level of 16.2 milligrams per deciliter (mg/dL) (3–5 mg/dL), a total bilirubin level of 29 μg/L (0–17 μg/L) with direct bilirubin of 10 μg/L (0–6 μg/L), an alkaline phosphatase (ALP) level of 642 units per liter (U/L) (35–110 U/L), gamma-glutamyl transferase (GGT) levels of 297 U/L (1–48 U/L), glutamic-pyruvic transaminase (GPT) levels of 301 U/L (1–31 U/L), and glutamic-oxaloacetic transaminase (GOT) levels of 279 U/L (1–31 U/L).

**Figure 1:**
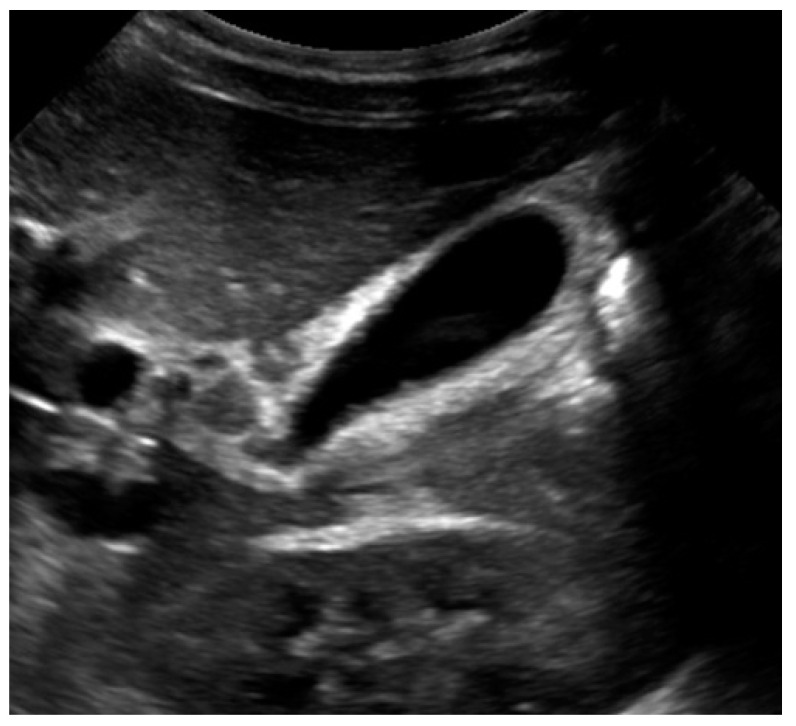
Abdominal ultrasonography showing a thickening of the gallbladder wall of 4.5 mm and pericholecystic edema.

Formal abdominal ultrasonography revealed a thickening of the gallbladder wall (4.5 mm) and pericholecystic edema (Figure 1). Distention of the gallbladder was not evident, and no stones or dilatation of the biliary tract were reported. Based on these findings, the diagnosis of acute acalculous cholecystitis was made and the patient was admitted to the pediatrics department for further investigation and treatment.

The patient was treated with fasting and intravenous rehydration. Her cardiovascular and respiratory function remained stable. Blood and urine cultures were negative. Serological tests, both heterophile antibody test and IgM antibodies against EBV capsid antigen, confirmed the diagnosis of infectious mononucleosis. Serological tests for hepatitis A, B, C, CMV, HIV, and *Toxoplasma gondii* were negative.

Two days after admission, the patient had a good clinical status, fever was resolved, enteral nutrition was restarted, and the abdominal pain had also disappeared. In the laboratory test on day 3, a total bilirubin (B) level of 19 μg/L with direct bilirubin of 7 μg/L, an alkaline phosphatase (ALP) level of 199 units per liter (U/L), gamma-glutamyl transferase (GGT) levels of 48 U/L, glutamic-pyruvic transaminase (GPT) levels of 19 U/L and glutamic-oxaloacetic transaminase (GOT) levels of 22 U/L were observed.

The patient was discharged on day 4 with a good clinical status. Two weeks later, a clinical control in the outpatient department revealed no clinical or laboratory abnormalities and an abdominal ultrasound revealed a total remission of the previous abnormal findings.

After 6 months of follow up, the patient remains asymptomatic.

## 3. Discussion

Epstein–Barr virus (EBV) infection can lead to infectious mononucleosis syndrome with the typical symptoms of fever, pharyngitis, lymphadenopathy, and hepatosplenomegaly. However, cholecystitis is not usually considered part of a primary EBV infection and ultrasound scan (USS) of the liver and gallbladder is not routinely performed [5]. In our case, we performed the USS in order to identify a possible inflammation of the appendix because abdominal pain on the right abdominal side.

Acute acalculous cholecystitis (AAC) is a gallbladder infection not related to gallstones. It is more frequently found in males and represents 5–10% of all cases of acute cholecystitis in adults. Cholecystitis in children is not common (Tsakayannis et al. estimated 1.3 pediatric cases for every 1000 cases in adults) and 30–50% of these patients are AAC [6]. To our knowledge, only a few cases of EBV-associated AAC have been described in pediatric patients (Table 1).

Among the age-related predisposing diseases, systemic infectious diseases such as EBV were the most common ones in middle childhood and adolescence [25].

The exact pathogenesis of EBV-associated AAC is unclear; EBV-associated hepatitis is recognized as an important cause of cholestasis, suggesting that acute infection may induce the bile stasis and subsequent gallbladder inflammation that led to the development of AAC [22].

In children, AAC occurs more frequently during the course of infectious diseases such as sepsis, gastroenteritis (including Vibrio cholerae, Salmonella spp, Shigella spp, and Giardia lamblia), pneumonia (especially due to Mycoplasma pneumoniae), cytomegalovirus primary infection, hepatitis A, Dengue, infection secondary to non-tuberculous mycobacteria, leptospirosis, Q-fever, Candida, malaria, and HIV infection. It has also been associated with the use of parenteral nutrition, post-surgery, extensive burns, and after trauma. In healthy patients such as the related case, this form of cholecystitis is even less frequent [6,26].

The diagnosis of AAC in children remains a clinical challenge, especially in critical patients. Clinical, laboratory, and imaging findings will finally lead to diagnosis. Clinical presentation has been reviewed, concluding that right upper quadrant abdominal pain and fever remains the most common symptoms. Sore throat and pharyngitis, cervical lymphadenopathy, abdominal tenderness, and Murphy’s sign are also common findings. Elevation of GGT, GOT, ALP, and bilirubin in various degrees was also reported [27]. After three days of hospital stay, our patient presented decreasing GGT, GPT and GOT values within the reference range. After two weeks of follow up, B and ALK were also normalized.

We performed a review of the abdominal sonographic findings in pediatric patients with EBV-associated AAC, and it has been related the sonographic gallbladder wall thickening greater than 3 mm, hepatomegaly, splenomegaly, increased periportal echogenicity, and periportal lymph nodes [28]. Pericholecystic fluid (halo) or subserosal edema, intramural gas or hydrops and the presence of echogenic bile have also been described. In uncertain cases, computed tomography and cholescintigraphy using 99mTc are reasonable imaging alternatives to establish the diagnosis [26,28].

In children with AAC due to EBV, medical treatment should be considered the management of choice; excellent recovery without surgical treatment is generally expected. The use of intravenous antibiotics is controversial, and emergency surgical intervention should only be considered if ultrasonographic abnormalities persist or worsen on follow-up examinations [23]. In our case, we proposed intravenous fluids, nonsteroidal anti-inflammatory drugs, and rest, with excellent recovery in two days.

The incidence of complications in AAC for EBV is low when prompt and accurate diagnosis and treatment are performed. If the diagnosis is delayed or incorrect, complications such as perforation and gangrene have been described. Graber et al. presented the case of a 15-year-old female who underwent laparoscopic cholecystectomy for EBV-associated AAC and died after surgery due to unexplained postoperative complications. Furthermore, poor predictors of AAC mortality include the presence of thrombocytopenia, anemia, gallbladder sludge, associated hepatitis, and sepsis plus hepatitis [24,29].

## 4. Conclusions

Despite AAC being a rare complication of infectious mononucleosis for EBV in children, a high index of suspicion is important for the diagnosis, especially in patients without a relevant past medical history. Our patient recovered fully with conservative treatment without antibiotics. Surgical intervention has not been described as the procedure of choice of ACC for EBV, and should be considered only in severe cases that are not responding to conservative therapy.

## Figures and Tables

**Table 1:** Cases of Epstein–Barr virus-associated acute acalculous cholecystitis in pediatric patients.

Author	Year	Age	Sex	Antibiotic	Surgery	Favorable Outcome
Yoshie et al. [7]	2004	15	F	-	No	Yes
Lagona et al. [8]	2007	4	F	No	No	Yes
Prassouli et al. [9]	2007	13	F	Yes	No	Yes
Gora-Gebka et al. [10]	2008	9	F	Yes	No	Yes
Gora-Gebka et al. [10]	2008	4	F	Yes	No	Yes
Pelliccia et al. [11]	2008	14	F	No	No	Yes
Attilakos et al. [12]	2009	5	F	Yes	No	Yes
Attilakos et al. [12]	2009	4	M	No	No	Yes
Arya et al. [13]	2010	16	F	Yes	No	Yes
Aydin Teke et al. [14]	2013	8	F	No	No	Yes
Fretzayas et al. [15]	2014	11	F	-	No	Yes
Fretzayas et al. [15]	2014	12	F	-	No	Yes
Kim et al. [3]	2014	10	F	Yes	No	Yes
Poddighe et al. [16]	2014	7	F	Yes	No	Yes
Suga et al. [17]	2014	6	F	Yes	No	Yes
Alkhoury et al. [18]	2015	15	F	No	No	Yes
Shah et al. [19]	2015	6	F	Yes	No	Yes
Branco et al. [20]	2015	16	M	Yes	No	Yes
Majdalani et al. [21]	2016	16	F	Yes	No	Yes
Say et al. [22]	2016	14	F	Yes	No	Yes
Rodà et al. [23]	2017	2	M	Yes	No	Yes
Graber et al. [24]	2018	15	F	Yes	Yes	No
Young et al. [4]	2019	14	F	Yes	No	Yes
Present case	2020	10	F	No	No	Yes

## Data Availability

Not applicable.
